# Exosomes in the f ield of reproduction: A scientometric study and visualization analysis

**DOI:** 10.3389/fphar.2022.1001652

**Published:** 2022-09-23

**Authors:** Yifeng Shen, Yaodong You, Kun Zhu, Chunyan Fang, Degui Chang, Xujun Yu

**Affiliations:** ^1^ Hospital of Chengdu University of Traditional Chinese Medicine, Chengdu, China; ^2^ TCM Regulating Metabolic Diseases Key Laboratory of Sichuan Province, Hospital of Chengdu University of Traditional Chinese Medicine, Chengdu, China; ^3^ School of Medicine and Life Sciences, Chengdu University of Traditional Chinese Medicine, Chengdu, China

**Keywords:** exosomes, reproduction, Citesmall space, VOSviewer, bibliometrics

## Abstract

**Background:** The diagnostic capabilities of exosomes in the field of reproductive biomedicine have attracted much attention. The aim of this scientometric study was to statistically and qualitatively assess the knowledge structure, hot issues, and research trends of papers about exosomes in the field of reproduction using visualization methods.

**Methods:** The Web of Science Core Collection was searched for studies on exosomes in the field of reproduction. We performed bibliometric and visual analyses using VOSviewer, CiteSpace, and Microsoft Excel.

**Results:** After database search, 1,011 articles were included, with number of studies being published every year continually increasing. These publications came from 61 nations or regions, with the US having the highest number. The University of Queensland was the main institution in which the research was conducted. The journal *Placenta* contained the highest number studies. There were 5,247 authors in total. Carlos Salomon had the highest number of papers with co-citations. Exosomes, extracellular vesicles, pregnancy, microRNAs, preeclampsia, placenta, microvesicles, gene expression, biomarkers, and first trimester were the most frequently used terms.

**Conclusion:** Exosome research is booming in reproductive biomedicine. Future studies will likely focus on exosomes as biomarkers in gamete formation and fertilization, pregnancy, and cancers associated with reproduction. In addition to focusing on fundamental research, we should concentrate on the application of the results and the investigation of exosomes in infertile patients.

## Introduction

Extracellular vesicles (EVs) are membrane-enclosed, phenotypically diverse particles that are released by practically all mammalian cell types. When cells internalize or come into contact with EVs, they might undergo phenotypic alterations. EVs can be divided into four subgroups according to their biogenesis, size, and biophysical properties: 1) apoptotic bodies/bleb (vesicles with a diameter of about 50–5,000 nm produced during apoptosis); 2) microvesicles (referred to ectosomes or microparticles; 100–1,000 nm); 3) exosomes (30–150 nm); and 4) oncosomes (1–10 μm). Microvesicles are EVs that bleb from the cell membrane directly, whereas exosomes are EVs that are produced when a cytoplasmic endosome containing several vesicles fuses with the cell membrane. Apoptotic bodies are EVs that are the leftovers of an apoptotic cell that has decomposed. Oncosomes are a newly discovered class that have been observed in cancer cells (Yanez-Mo et al., 2015; Yang et al., 2017). The current research focuses more on the exosome.

Exosomes were first identified in 1983 during the *in vitro* maturation of sheep reticulocytes (Pan and Johnstone, 1983). Exosomes are nanovesicles include lipid bilayers, proteins, and genetic material. Exosomes were once classified as “cell trash” and referred to as “membrane fragments.” With the advancement of science and research methodologies, it has been discovered that exosomes serve as a form of intercellular communication in numerous physiological and pathological processes and are involved in the transport of biomolecules. Exosomes play a role in maintaining the body’s homeostasis and in the movement of biomolecules, and they carry bioactive materials such as proteins, lipids, enzymes, and nucleic acids such as RNA and DNA (Kalluri and Lebleu, 2020).

Exosomes are nanoscale entities seen in sperm, epididymal fluid, endometrium, and follicular fluid. They have been shown to influence processes in both the male and female reproductive systems, including gametogenesis, acrosomal response, sperm capacitation, and embryo implantation (Kowalczyk et al., 2022). Extracellular vesicles (EVs) in the seminal fluid of males are associated with posttesticular sperm maturation, including the acquisition of sperm mobility and the reduction of oxidative stress. EVs in the follicular fluid of women have been found to include miRNAs with putative functions in follicular development, continuation of oocyte meiosis, steroidogenesis, and the avoidance of polyspermy after fertilization. EVs were also found in the medium of cultured embryos, suggesting that EVs released from embryos and the uterus may facilitate embryo–endometrium communication during implantation (Machtinger et al., 2016). Exosomes are composed of the same molecules as their parent cells and are released by both healthy and diseased cells. Consequently, exosomes can reflect the physiology of cells. In addition, due to the movement of biomolecules, they participate in intercellular communication and can be used as biomarkers for a variety of disorders, including ovarian and endometrial cancer (Wang et al., 2021a). The identification of exosomes as biomarkers could aid in the comprehension of genital dysfunction and infertility diseases.

Over the 40 years since exosomes were first proposed, there has been progress in research on exosomes in reproductive biomedicine regarding their diagnostic, therapeutic, and prognostic potential, but some issues remain to be explored. Here, we address exosomes as mediators and messengers in reproductive biology to show the impact exosomes have on placentation and pregnancy problems, the function of exosomes in reproduction, the significance of exosomes in the male reproductive system, and their involvement in the cross-communication between the female and the embryo. It is important to underline the function of exosomes in reproductive biomedicine and therapeutic medicine as well as the function of microRNAs in embryo implantation. The potential applications of exosomes in reproductive disease should be studied in the future.

Bibliometrics and visual analysis are effective tools for integrating information and enhancing comprehension of the research process. In this review, we first applied scientometric approaches and extensive visualization tools to examine the literature on exosomes in the field of reproductive biomedicine in an effort to shed light onto the existing state of affairs and future prospects in this field.

## Materials and methods

### Sources of data and search strategies

**FIGURE 1:**
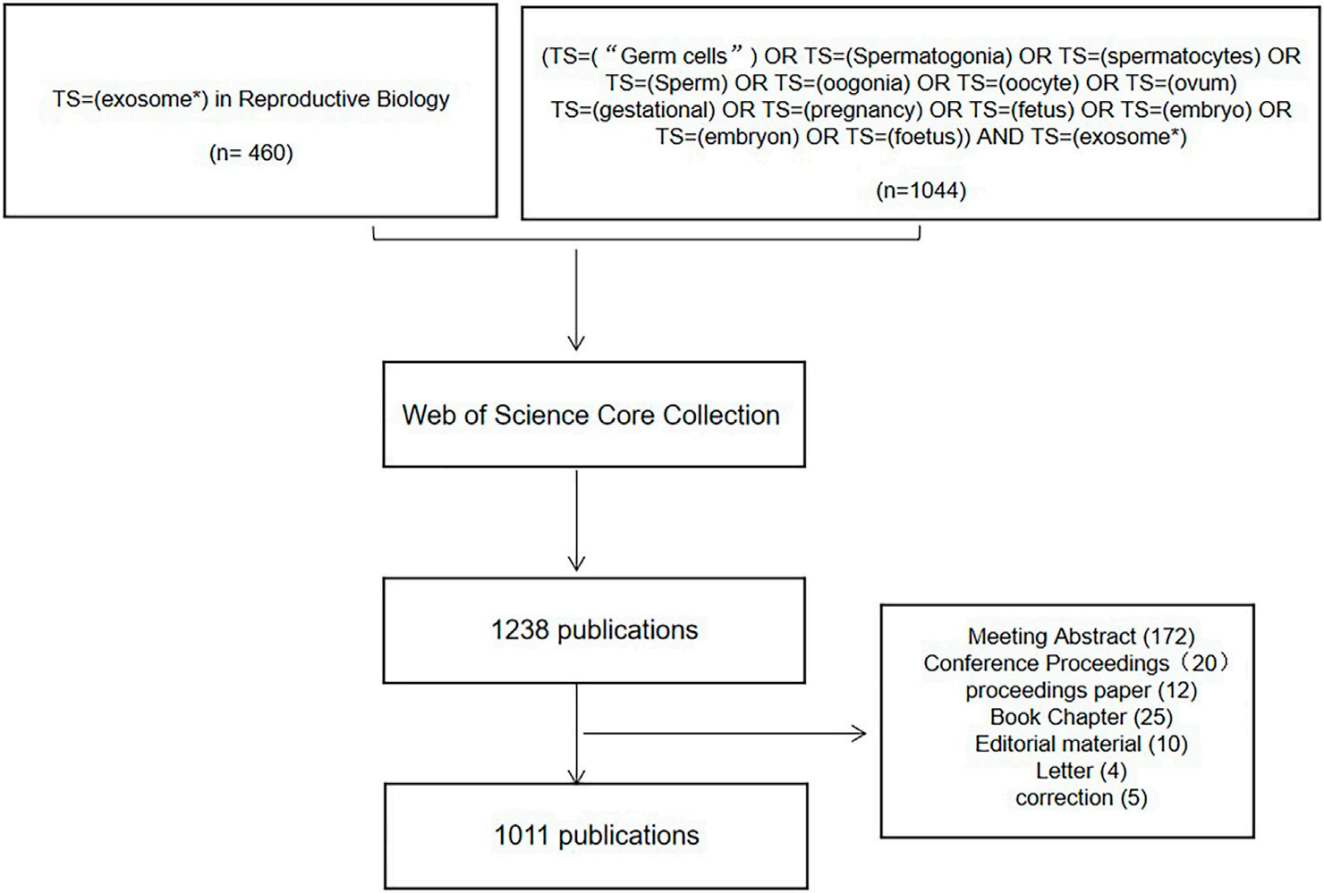
Flowchart of search strategy and included studies.

Through the Web of Science (WOS), the Science Citation Index Expanded (SCI-E) core database’s connected papers from the commencement of the databases until 2022. Data was downloaded within 1 day on 30 June 2022. The search term “exosome*” with the category “reproduction research” was used to refine the search results. To avoid the loss of literature owing to WOS subject classification, we additionally searched using reproductive-related keywords and “exosomes,” and the results obtained were also included in this study. The search included reviews and original research publications. The language setting for articles was English. Data on titles, authors, publication dates, countries/regions, institutions, journals, keywords and citation counts were all exported and saved as plain text files for the retrieved eligible publications. The authors browsed to select studies focusing only on exosomes. Studies focusing on apoptotic bodies and microvesicles were excluded from the study. A total of 1,011 items that matched the search criteria were located and further evaluated (Figure 1).

### Data collection and analysis

The authors collected and screened the WOSCC (Web of Science Core Collection Web of Science Core Collection) raw data independently. VOSviewer (version 1.6.18), CiteSpace (version 6.1.R3), and Excel (version 2020) were used to analyze the data. Disagreements were resolved through discussion. Using CiteSpace and VOSviewer, we explored journal associations, evaluated collaborative teams across nations, institutions, and authors, built visualization maps, recorded keywords, and identified cocited authors/references.

## Results

### Publications

**FIGURE 2:**
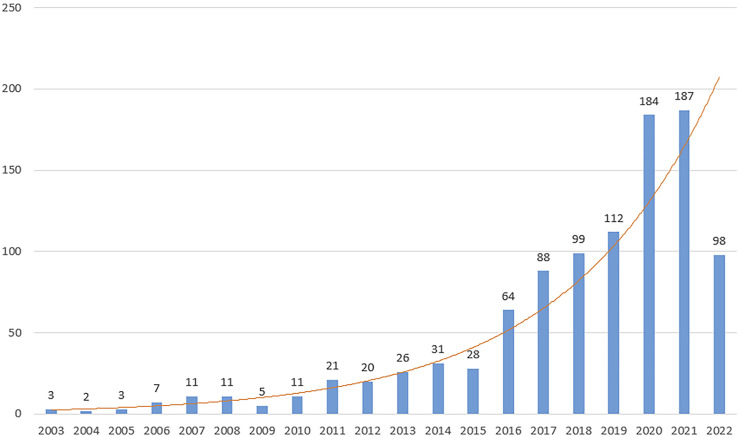
Trends in studies of exosomes published among reproduction studies. The annual number of publications on exosomes in reproduction research from 2003 to 2022.

There were 1,011 articles that met the retrieval criteria. The total number of publications published annually is presented in Figure 2, with the trend spanning from three papers in 2003 to 187 papers in 2021. Since 2016, there has been a substantial increase in the number of published research publications. The annual average number of articles published was 51. As of 30 June 2022, 98 articles had been published.

### Countries and institutes

**FIGURE 3:**
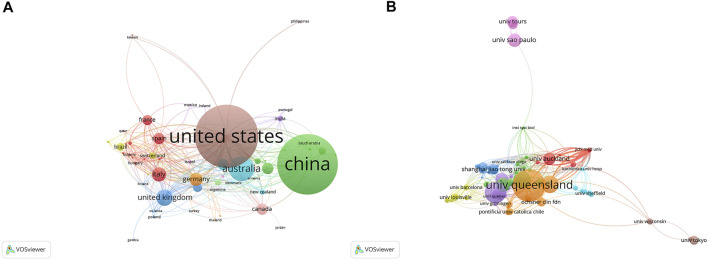
The distribution of countries and institutions publishing research on exosomes in reproduction. **(A)** The network map of countries/regions publishing research on exosomes in reproduction. **(B)** The network map of institutes involved in research on exosomes in reproduction.

Studies addressing exosomes in reproduction have been published by 61 regions/countries. These regions/countries possessed a wide variety of collaborative teams (Figure 3A). Among the top ten regions/countries (Table 1) engaged in research on exosomes in reproduction, the United States ranked first with 264 articles, followed by China (253), Australia (105), the United Kingdom (69), and Italy (60). More than 99 institutes were performing research on reproduction exosomes. There were numerous partnerships among the institutes (Figure 3B). The top 10 institutes accounted for approximately 37.82% of all publications (Table 1). The University of Queensland topped the list, followed by the University of Oxford, the University of Concepcion, the University of Texas Medical Branch, and the University of Pittsburgh.

**TABLE 1:** Distribution of publications on exosomes in reproduction across different countries and institutions.

Country/region	Count	Percent (%)	Institute	Count	Percent (%)
United States	264	17.78	Univ Queensland	61	8.64
China	253	17.04	Univ Oxford	36	5.10
Australia	105	7.07	Univ Concepcion	28	3.97
United Kingdom	69	4.65	Univ Texas Med Branch	28	3.97
Italy	60	4.04	Univ Pittsburgh	21	2.97
Germany	55	3.70	Univ Melbourne	20	2.83
Canada	48	3.23	Univ Sao Paulo	20	2.83
Japan	48	3.23	Nanjing Med Univ	19	2.69
Spain	48	3.23	Univ Auckland	18	2.55
Chile	47	3.16	Univ Tours	16	2.27

### Journals

**FIGURE 4:**
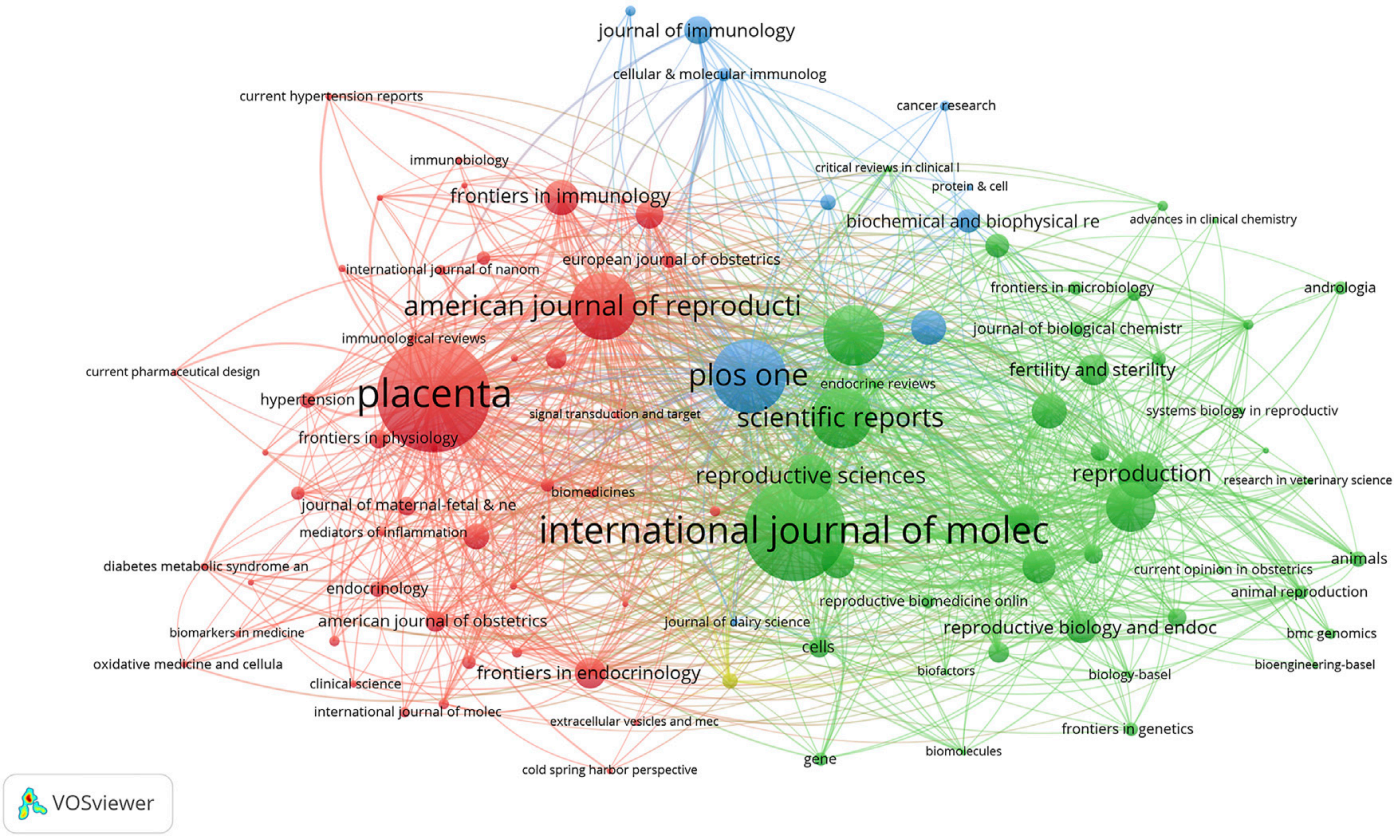
The distribution of journals publishing research on exosomes in reproduction.

The majority of studies on reproduction and exosomes were published in various publications between 2003 and 2022, with the top 10 journals listed in Table 2 and Figure 4. With 101 articles published, *Placenta* was the journal with the highest number of such articles. In 2021, the impact factors of these journals ranged from 4.2 to 8.1, with *Frontiers in Immunology* having the highest impact factor and *Theriogenology* the lowest. According to the JCR partition analysis, Q1 constituted 40% of this ranking, Q2 constituted 50%, and Q3 constituted 10%.

**TABLE 2:** Top 10 journals with the largest number of publications on exosomes in reproduction.

Journals	Documents	2021 impact factor	2021 JCR partition
Placenta	56	5.50	Q2
International Journal of Molecular Sciences	49	6.00	Q1
Plos One	33	5.30	Q2
American Journal of Reproductive Immunology	29	5.80	Q2
Biology of Reproduction	26	5.60	Q2
Scientific Reports	26	7.10	Q2
Theriogenology	20	4.20	Q1
Reproduction	19	6.00	Q1
Reproductive Sciences	18	4.50	Q3
Frontiers in Immunology	13	8.10	Q1

### Authors

**FIGURE 5:**
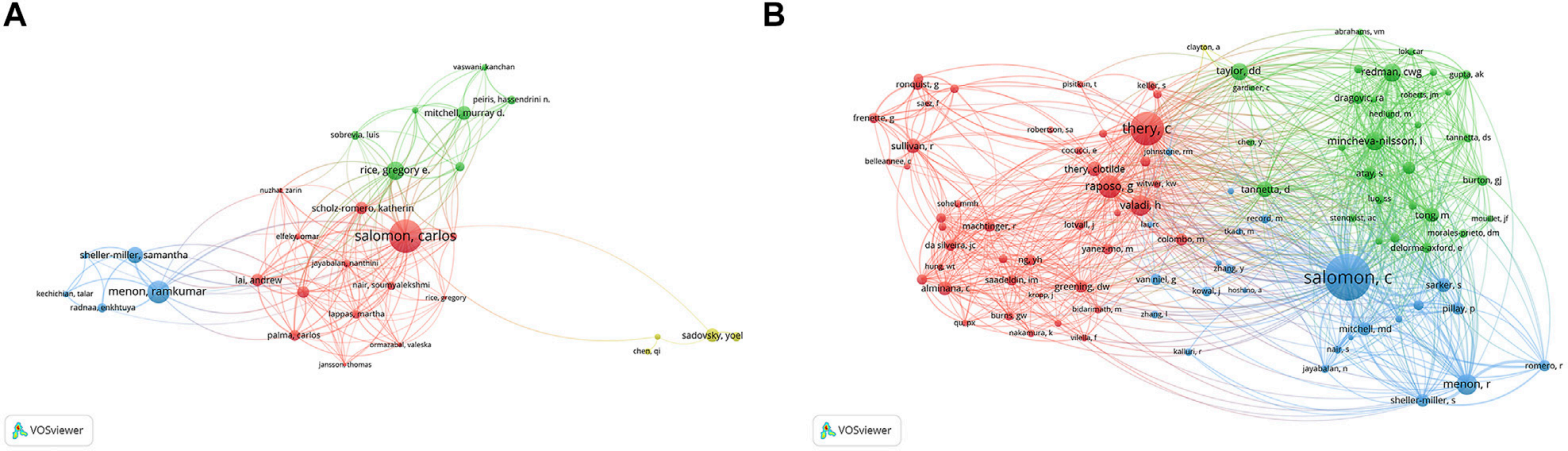
The distribution of authors publishing research on exosomes in reproduction. **(A)** Collaboration network among the authors. **(B)** co-citations network among the authors.

A total of 5,247 authors were involved in exosome and reproduction research. The authors’ collaborative network is depicted in Figure 5A. Salomon, Carlos (43 publications) was first among the top 10 contributing authors (Table 3), followed by Menon, Ramkumar (27 publications), Rice, Gregory E. (21 publications), Sheller-Miller, Samantha (18 publications), and Mitchell, Murray D. (15 publications). The data on author citations were shown in a co-citation network (Figure 5B). Salomon, C (587 co-citations) was first among the top 10 cocited authors (Table 3), followed by Thery, C (397 co-citations), Raposo, G (235 co-citations), Menon, R (220 co-citations), and Valadi, H (211 co-citations).

**TABLE 3:** Top 10 authors and co-cited authors on exosomes in reproduction.

Author	Count	Co-cited author	Count
Salomon, Carlos	43	Salomon, C	587
Menon, Ramkumar	27	Thery, C	397
Rice, Gregory E	21	Raposo, G	235
Sheller-Miller, Samantha	18	Menon, R	220
Mitchell, Murray D	15	Valadi, H	211
Sadovsky, Yoel	14	Mincheva-Nilsson, L	189
Lai, Andrew	13	Redman, Cwg	182
Scholz-Romero, Katherin	13	Taylor, Dd	171
Guanzon, Dominic	12	Tannetta, D	149
Palma, Carlos	11	Thery, Clotilde	140

### Citations

**FIGURE 6:**
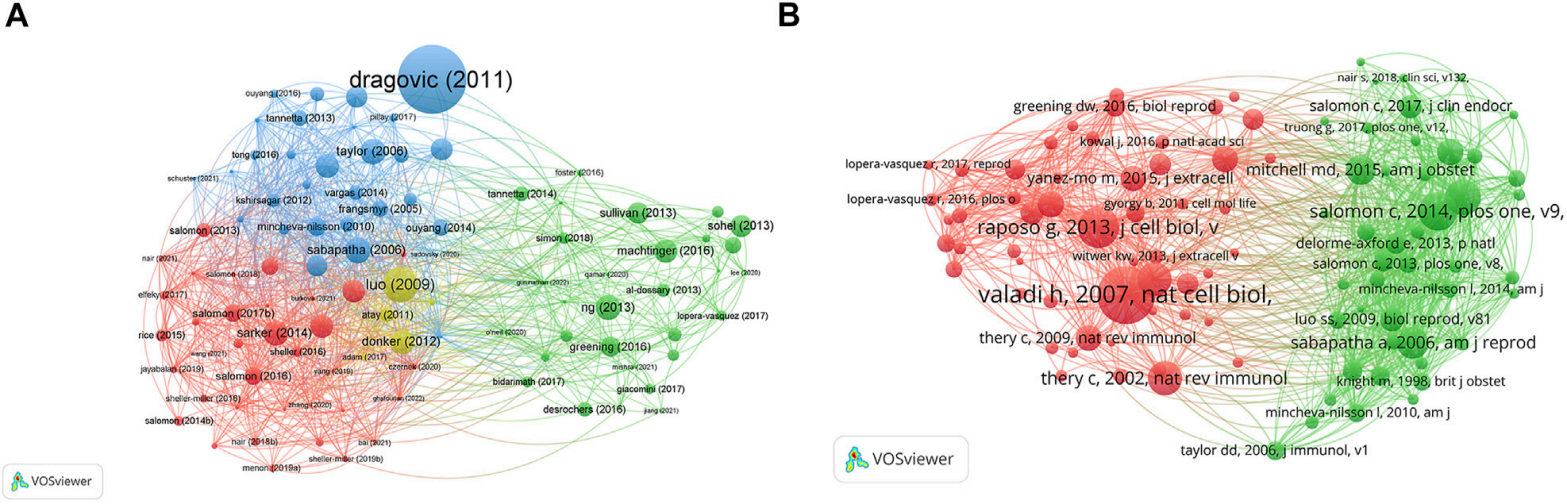
The distribution of citations pertaining to research on exosomes in reproduction. **(A)** Citation analysis of documents. **(B)** co-citations analysis of documents.

The top 10 studies with the highest citations are included in Table 4, which indicated the number of citations of the documents (Figure 6A), and the range of citation numbers was from 782 to 199. “Sizing and phenotyping of cellular vesicles using nanoparticle tracking analysis” (2011), published by Dragovic RA, had the highest number of citations, at 782 citations; “Human villous trophoblasts express and secrete placenta-specific microRNAs into maternal circulation *via* exosomes,” published by Luo SS (2009), which had the second highest number, at 344 citations; Pregnancy-associated exosomes and their modulation of T cell signaling” (2006) by Taylor DD, which had 229 citations. To analyze the citations of the documents, co-citations analysis of the cited references was performed (Figure 6B; Table 5).

**TABLE 4:** Top 10 documents in citation analysis of publications on exosomes in reproduction.

Rank	Title	First author	Source	Publication year	Total citations
1	Sizing and phenotyping of cellular vesicles using Nanoparticle Tracking Analysis	Dragovic R. A.	Nanomedicine	2011	782
2	Human villous trophoblasts express and secrete placenta-specific microRNAs into maternal circulation via exosomes	Luo S. S.	Biol Reprod	2009	344
3	Pregnancy-associated exosomes and their modulation of T cell signaling	Taylor D. D.	J Immunol	2006	229
4	Specific isolation of placenta-derived exosomes from the circulation of pregnant women and their immunoregulatory consequences	Sabapatha A.	Am J Reprod Immunol	2006	226
5	Endometrial exosomes/microvesicles in the uterine microenvironment: a new paradigm for embryo-endometrial cross talk at implantation	Ng YH	PLoS One	2013	225
6	Placenta-derived exosomes continuously increase in maternal circulation over the first trimester of pregnancy	Sarker S.	J Transl Med	2014	223
7	The expression profile of C19MC microRNAs in primary human trophoblast cells and exosomes	Donker R. B.	Mol Hum Reprod	2012	212
8	A gestational profile of placental exosomes in maternal plasma and their effects on endothelial cell migration	Salomon C.	PLoS One	2014	205
9	Extracellular vesicles: roles in gamete maturation, fertilization and embryo implantation	Machtinger R.	Hum Reprod Update	2016	204
10	Human placenta expresses and secretes NKG2D ligands via exosomes that down-modulate the cognate receptor expression: evidence for immunosuppressive function	Hedlund M.	J Immunol	2009	199

**TABLE 5:** Top 10 documents in co-citation analysis of publications on exosomes in reproduction.

Rank	Title	First author	Source	Publication year	Total citations
1	Exosome-mediated transfer of mRNAs and microRNAs is a novel mechanism of genetic exchange between cells	Valadi H.	Nat Cell Biol	2007	207
2	Extracellular vesicles: exosomes, microvesicles, and friends	Raposo G.	J Cell Biol	2013	148
3	A gestational profile of placental exosomes in maternal plasma and their effects on endothelial cell migration	Salomon C.	PLoS One	2014	138
4	Placenta-derived exosomes continuously increase in maternal circulation over the first trimester of pregnancy	Sarker S.	J Transl Med	2014	129
5	Exosomes: composition, biogenesis and function	Théry C.	Nat Rev Immunol	2002	122
6	Isolation and characterization of exosomes from cell culture supernatants and biological fluids	Théry C.	Curr Protoc Cell Biol	2006	122
7	Specific isolation of placenta-derived exosomes from the circulation of pregnant women and their immunoregulatory consequences	Sabapatha A.	Am J Reprod Immunol	2006	113
8	Placental exosomes in normal and complicated pregnancy	Mitchell M. D.	Am J Obstet Gynecol	2015	107
9	Endometrial exosomes/microvesicles in the uterine microenvironment: a new paradigm for embryo-endometrial cross talk at implantation	Ng YH	PLoS One	2013	94
10	Gestational Diabetes Mellitus Is Associated With Changes in the Concentration and Bioactivity of Placenta-Derived Exosomes in Maternal Circulation Across Gestation	Salomon C.	Diabetes	2016	94

### Keywords

**FIGURE 7:**
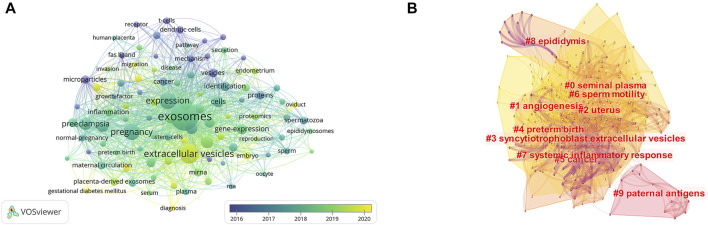
The distribution of citations pertaining to research on exosomes in reproduction. **(A)** Co-occurrence analysis of keywords. **(B)** Keyword Cluster Analysis.

**FIGURE 8:**
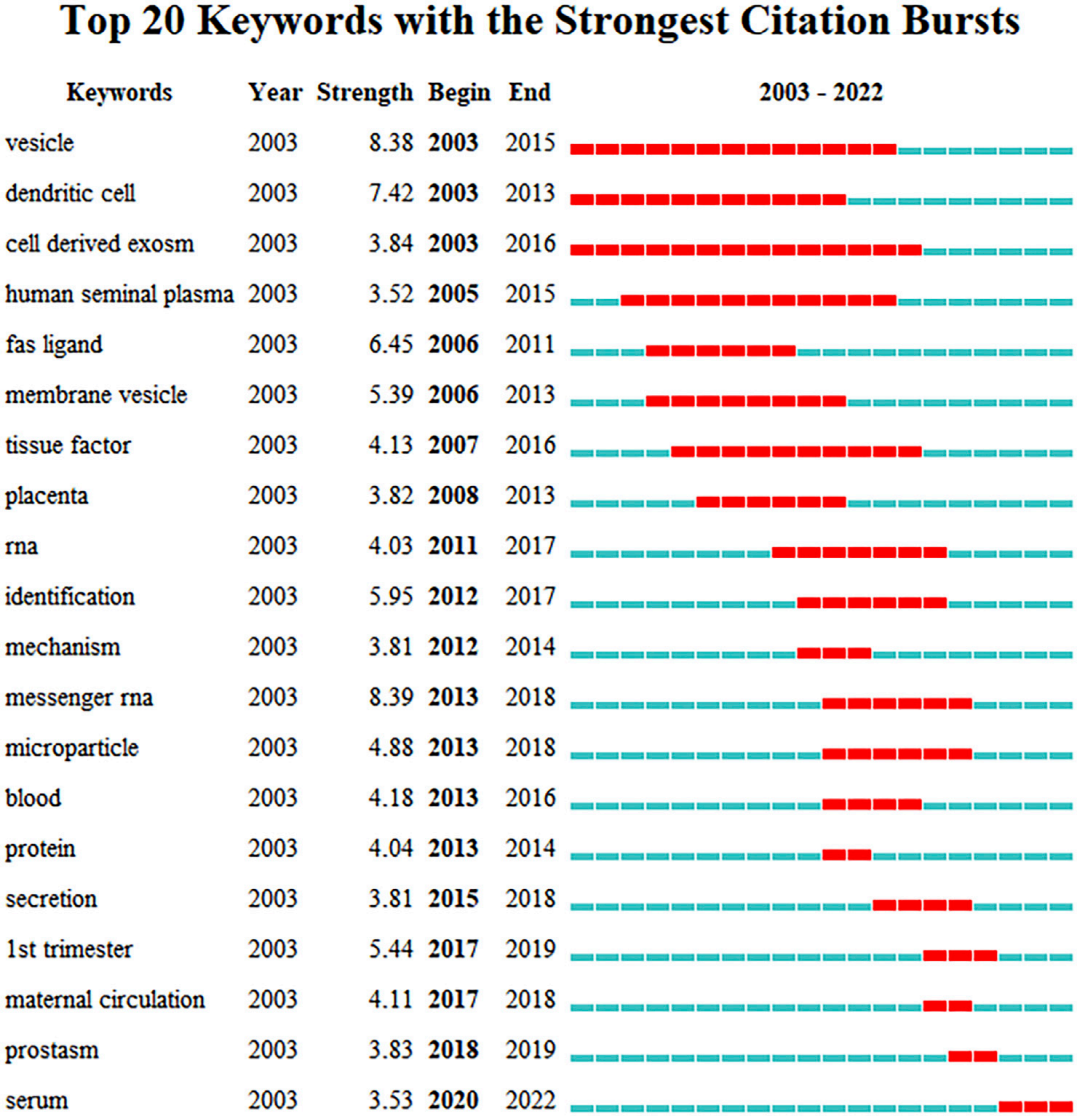
Burst word analysis of keywords to research on exosomes in reproduction.

There were 4,408 keywords overall, 417 of which were used in at least five documents. The colors in overlay depiction in Figure 7A represent the average publication year of the discovered keywords. The majority of the keywords, with greener or yellower hues, were released after 2016. Exosomes, extracellular vesicles, pregnancy, microRNAs, preeclampsia, placenta, microvesicles, gene expression, biomarkers, and first trimester were high-frequency keywords. Keywords can be divided into the following categories: seminal plasma, angiogenesis, uterus, syncytiotrophoblast extracellular vesicles, preterm birth, cancer, sperm motility, systemic inflammatory response, epididymis, and paternal antigens (Figure 7B; Table 6). Terms with a high citation explosion were analyzed by CiteSpace, as shown in Figure 8.

**TABLE 6:** Top 30 keywords of documents on exosomes in reproduction.

No.	Keywords	Count	No.	Keywords	Count	No.	Keywords	Count
1	Exosomes	602	11	Gene-expression	79	21	Proteins	53
2	Extracellular vesicles	286	12	*In-vitro*	79	22	Microparticles	52
3	Expression	212	13	Identification	68	23	Mirna	51
4	Pregnancy	208	14	Biomarkers	65	24	Maternal circulation	49
5	Micrornas	119	15	1st trimester	60	25	Microrna	49
6	Preeclampsia	117	16	Vesicles	58	26	Placenta-derived exosomes	46
7	Exosome	116	17	Biogenesis	55	27	Angiogenesis	45
8	Placenta	112	18	Cancer	55	28	Spermatozoa	44
9	Microvesicles	105	19	Protein	54	29	Dendritic cells	43
10	Cells	98	20	Inflammation	53	30	Activation	42

## Discussion

### General trends

We used bibliometric analyses and network visualizations to characterize the current landscape of exosomes in the reproduction research field, analyzing the contributions of countries, institutions, journals, and authors to this emerging field, and predicting research hot topics that will remain of interest in the coming years. Since the field’s foundation in 2003, annual publishing output has steadily increased, with the last 7 years accounting for 83 percent of all recognized publications. The United States now has the highest number of publications on exosomes and reproduction and the highest ranking for coauthorship analysis by nation. These results indicate that the United States may have a significant impact on the direction of research in this field and that its collaboration is among the strongest in the world. Additionally, the number of studies conducted in China, Australia, and the United Kingdom has expanded substantially. The most productive institution, the University of Queensland, topped the university’s coauthorship analyses, suggesting substantial collaboration with other institutions.

### Influential authors and studies

Salomon, Carlos of The University of Queensland, is a pioneer in the field of exosome reproduction studies, with the highest number of publications and coauthorship analyses. He is a nationally and internationally recognized researcher in the field of EVs who specializes in reproductive biology (specifically in pregnancy and its complications). Salomon, Carlos has established and directs the Exosome Biology Laboratory that conforms to ISO standards at the University of Queensland Centre for Clinical Research, where human exosomes can be isolated and characterized and their role clarified to evaluate their clinical utility as biomarkers of disease and therapeutic interventions (Reiner et al., 2017). Using gold-loaded ferric oxide nanocubes (Au-NPFe_2_O_3_NC), a highly sensitive, quick, and affordable assay, Carlos Salomon and his research team envisioned a straightforward method for the direct isolation and subsequent detection of a specific population of exosomes. This method could be useful in quantifying specific populations of exosomes for a variety of clinical applications, with a focus on pregnancy complications (Boriachek et al., 2019).

Ramkumar Menon of the University of Texas Medical Branch provided evidence that exosomes operate as paracrine labor and delivery mediators (Sheller et al., 2016; Sheller-Miller et al., 2019). During pregnancy, exosomal cargo can be transported systemically from fetal to maternal uterine tissue, supporting the notion that fetal signals can be conveyed by exosomes (Sheller-Miller et al., 2016). Pregnancy problems are often caused by abnormal exosomal signaling by placental cells, which is a clinically significant biomarker of pregnancy issues. Pregnancy complications are characterized by incomplete spiral arteries remodeling (Truong et al., 2017).

In the context of reproduction, the vast majority of high-impact exosomal research (high number of citations and co-citations) focuses on the function and expression of exosomes in the uterus during pregnancy. The quantity of exosomes in maternal plasma increases dramatically during pregnancy, and these exosomes are bioactive (Salomon et al., 2014). Exosomes from the placenta have emerged as novel immunological regulators in maternal immune tolerance (Hedlund et al., 2009). Placental exosomes inhibit T-cell signaling components (Sabapatha et al., 2006). Exosomes released from human chorionic villi and then enter the mother’s bloodstream, are capable of secreting miRNAs extracellularly (Luo et al., 2009). Exosomes containing particular miRNAs are present in the milieu where embryo implantation takes place and may contribute to the endometrial–embryo communication required for this process (Ng et al., 2013). Throughout the first trimester of a typical, healthy pregnancy, the number of exosomes in the maternal plasma increases dramatically as the pregnancy progresses. Early screening of women at risk of pregnancy difficulties with exosomes would allow for the development and evaluation of appropriate intervention methods to limit acute unfavorable sequelae (Sarker et al., 2014). Pregnancies affected by gestational diabetes and preeclampsia have been linked to alterations in the release of placenta- and non-placenta-derived exosomes and their concentration in maternal plasma, composition, and bioactivity (Mitchell et al., 2015). The production of proinflammatory cytokines from endothelial cells is considerably boosted by exosomes isolated from gestational diabetes mellitus pregnancies (Salomon et al., 2016).

### Hotspots and frontiers

In the co-occurrence network maps, which were clustered by topic area or publication date, the current hot themes and future prospects in reproduction research involving exosomes were highlighted.

### Extracellular vesicles effects on gamete development and fertilization

MicroRNAs are among the cargo of human follicular fluid (FF) exosomes and are important in the regulation of follicle maturation (Santonocito et al., 2014). MiR-17, miR-92a, and miR-145 are the principal microRNA candidates in FF that regulate oocyte development (Inoue et al., 2020). Exosomes isolated from FF are able to influence members of the TGFB/BMP in mares (Da et al., 2014) and EGFR/MAPK in canine species (Lee et al., 2020) signaling pathways in granulosa cells and may regulate follicle and oocyte development. Cumulus cell expansion is necessary for fertilizable oocyte ovulation; plasma-derived EVs are capable of inducing cumulus cell expansion and oocyte maturation (Javadi et al., 2022). Porcine oviductal EVs could be used to increase sperm viability during sperm handling and improve IVF (*in vitro* fertilization) outcomes (Alcantara-Neto et al., 2020).

Sperm can still absorb vesicle-derived cargo after ejaculation; sperm motility and the capacity to undergo capacitation can be enhanced by exosomal transfer; and male tract exosomes influence the quality of sperm (Murdica et al., 2019a). Exosomes of normozoospermic (NSP) males enhance sperm motility and fast capacitation, but exosomes of men with severe asthenozoospermia (SA) lack these effects (Murdica et al., 2019b). Compared to normozoospermic males, oligoasthenozoospermic subfertile men have significantly higher expression levels of miR-765 and miR-1275 and significantly lower expression levels of miR-15a (Abu-Halima et al., 2016). MiR-539-5p and miR-941 expression values are mentioned as being helpful for predicting the presence of residual spermatogenesis in people with severe spermatogenic disorders with good diagnostic accuracy. MiR-31-5p expression values in exosomes from semen as a predictive biomarker test for the origin of azoospermia with high sensitivity and specificity have also been described (Barcelo et al., 2018). Exosomes secreted by Sertoli cells may pass the blood–testis barrier and aid Leydig cells in survival from a murine model (Ma et al., 2022). ExATP (extracellular adenosine triphosphate) produced in seminal plasma exosomes may delicately regulate mitochondrial metabolism to regulate sperm motility from a porcine model (Guo et al., 2019).

Exosomes in seminal plasma appear to interact not only with spermatozoa but also with cells from the female reproductive tract, altering their gene expression and influencing the female immunological response to semen (Jodar, 2019). Seminal plasma (SP) contains vast quantities of exosomes that are deposited in the female vaginal tract following insemination. Seminal exosomes (SEs) are ingested by endometrial stromal cells (eSCs) and trigger them to generate cytokines implicated in the immunology of embryo implantation, IL-6 and IL-8 in humans (Paktinat et al., 2019). The conditioned media produced from endometrial stromal cells exposed to seminal EVs induces IL-1 alpha and IL-6 production by macrophages, but IL-10 secretion is decreased (Paktinat et al., 2021). Defective chromatin packaging and histone removal in spermatozoa lead to inappropriate expression of paternal genes, which in turn results in aberrant embryo development in humans (Jena et al., 2021).

### Exosomes during pregnancy

EV protein cargos are implicated in biological processes linked to endometrial receptivity, embryo implantation, and early embryo development, providing support for the hypothesis of an EV-mediated communication system between the embryo and maternal endometrium. The proteins that have been identified *in vitro* studies of human cell lines may serve as novel biomarkers of ER and implantation success (Segura-Benitez et al., 2022). Endometrial epithelial exosomes promote embryo growth, development, and implantation, whereas the soluble secretome has a selective effect on mouse embryo outgrowth (Gurung et al., 2020). Exosomes secreted by the uterus during the early luteal phase may play crucial roles in the development of somatic cell nuclear transfer (SCNT) embryos from a bovine model (Qiao et al., 2018). The ability of EVs produced from trophoblasts to modulate endometrial gene expression may have implications for embryo–maternal communication during implantation in human cell lines (Godakumara et al., 2021). Trophoblast-derived exosomes induce dose-dependent increases in monocyte motility and cell contact-independent increases in the generation of proinflammatory cytokine/chemokine profiles (Godakumara et al., 2021).

Recurrent implantation failure (RIF) is defined as multiple embryo transfers without pregnancy. RIF-endometrial EVs may have a role in RIF etiology. The miRNA 6131 decreases HTR8/SVneo cell proliferation and invasion (Liu et al., 2021a). The high expression of sHLA-G (tot) and sHLA-G (EV), along with the presence of the 14-bp deletion allele, may contribute to implantation failure (Nardi et al., 2016). Exosome-mediated transport of placenta-associated microRNAs to maternal immune cells modulates the expression of target genes in recipient cells (Kambe et al., 2014). Exosomes are crucial transmitters between the fetus and mother, and they can traverse the placenta. Exosomes that originate from the fetus and are released under conditions associated with term labor can induce parturition-related alterations in the uterine tissues of the mother. In murine models, exosomes carrying inflammatory payloads can cause preterm birth (Menon and Shahin, 2021). Exosomes generated from fetal cells stimulate inflammation in uterine and cervical tissues to facilitate labor and delivery (Tantengco et al., 2021). Antiphospholipid antibodies (aPLs) are autoantibodies that cause pregnancy abnormalities; aPLs modify the cargo of placental EVs, thereby increasing the number of danger signals. These EV cargo modifications may explain how aPL contributes to the increased risk of recurrent preeclampsia and stillbirths (Tsai et al., 2022). Adding oviductal fluid EVs from the isthmus to the *in vitro* culture of cow embryos improves embryo growth and quality (Lopera-Vasquez et al., 2017).

Exosome trafficking within the placental microenvironment may connect these nanovesicles to placental interface organization, fetal tolerance, virus protection, and possibly mother–fetus communication (Record, 2014). In affluent nations, human CMV congenital infection is the main nongenetic cause of fetal deformity. There are currently no pregnancy-safe antivirals available. Placental trophoblast cells produce exosomes carrying microRNA from the chromosome 19 microRNA cluster (C19MC) that confer viral resistance on recipient cells (Hamilton et al., 2021). Secretion of the FasL form linked with exosomes may be one method by which the placenta fosters an immunological privilege status (Frangsmyr et al., 2005). Exosomes are immunosuppressive, downregulating maternal immunity in multipotent ways. Syncytiotrophoblast-derived microvesicles/microparticles have proinflammatory, immunologically activating, and procoagulant actions on the maternal immune system (Mincheva-Nilsson and Baranov, 2014). Pregnancy problems such as preeclampsia, premature birth, and gestational diabetes mellitus can arise if the placenta does not adjust to the changing environment during early pregnancy (Yang et al., 2019). Preeclampsia is a hypertensive condition of pregnancy characterized by proteinuria and/or organ failure and new-onset hypertension, and it is the primary cause of maternal morbidity and mortality (Schuster et al., 2021).Multiple differently expressed lncRNAs were discovered in exosomes produced by placental tissues of preeclampsia patients, implying that they may be involved in the incidence and progression of preeclampsia (Gong et al., 2021). Exosomes in preeclampsia are connected to aberrant amounts of soluble fms-like tyrosine kinase-1 (sFlt-1), soluble endoglin (sEng), placental growth factor (PlGF) (Wang et al., 2021b), hypoxia inducible factor-1 alpha (HIF-1 alpha) (Verma et al., 2018), 3-hydroxy-3-methylglutaryl-CoA synthase 1 (HMGCS1) (Ying et al., 2021), miR-486-5p (Taga et al., 2022) and placental protein 13 (PP13) (Sammar et al., 2018). Exosomes generated from mesenchymal stem cells have been shown to have anti-cancer properties. These exosomes may mend the pathophysiology of preeclampsia by suppressing extravillous trophoblast apoptosis and promoting these cells’ invasive capacity (Matsubara et al., 2021).

### Exosomes in reproduction-related cancer and other diseases

The death rate associated with ovarian cancer is the highest among gynecologic cancers. Exosomal proteins and lipids may have utility in early ovarian cancer detection (Matsubara et al., 2021). Plasma-derived exosomal miR-4732-5p (Liu et al., 2021b), miR-205 (Zhu et al., 2022), serum exosomal miR-34a (Maeda et al., 2020), miR-1290 (Kobayashi et al., 2018), miR-1307 and miR-375 (Su et al., 2019) may be promising candidate biomarkers for diagnosing ovarian cancer. MiR-130a in exosomes is the primary factor inducing angiogenic signaling in chemoresistant ovarian cancer cells (Li et al., 2021). MiR-200b is increased in plasma-derived exosomes and acts as an oncogene in ovarian cancer by increasing macrophage M2 polarization (Li et al., 2021). The migration and invasion of ovarian cancer cells with low metastatic potential are promoted by exosome-mediated transfer of CD44 from ovarian cancer cells with high metastatic potential (Shen et al., 2021). Most patients with cervical cancer are susceptible to acquiring cisplatin (DDP) and radiation resistance. Circ 0074269 was overexpressed in the exosomes of DDP-resistant cervical cancer cells and was capable of being administered by exosomes. In DDP-resistant cervical cancer cells, silencing circ 0074269 increased DDP sensitivity, suppressed proliferation, reduced migration, and triggered death. MiR-1323 is transported *via* exosomes released by cancer-associated fibroblasts and can modulate cellular processes, consequently influencing radioresistance or radiosensitivity (Fang et al., 2022). Overexpression of miR-192-5p in tumor-associated macrophage-derived exosomes can effectively inhibit the growth of endometrial cancer (Wang et al., 2022a).

Endometriosis is a chronic, estrogen-dependent gynecological condition characterized by nonmenstrual pelvic pain, infertility, and the extrauterine development of endometrial-like glands and stroma. Long noncoding RNAs, microRNAs, and proteins involved in histone modification, angiogenesis, and immunological regulation have been detected in peritoneal fluid and endometrial cell exosomes (Freger et al., 2021). By stimulating neuroangiogenesis, exosomes may play a crucial role in endometriosis (Freger et al., 2021). The characterization of endometriosis-specific exosomes such as miR-214-3p (Zhang et al., 2021a), extracellular vesicular Legumain pseudogene 1 (EV-LGMNP1) (Sun et al., 2022) and actin filament associated protein 1-antisense RNA 1 (AFAP1-AS1) (Wang et al., 2022b) could open up new diagnostic and investigative pathways for treating endometriosis (Nazri et al., 2020). Fibrosis is the principal pathogenic characteristic of endometriosis. miR-214 has important functions in fibrotic illness. Endometriosis patients’ serum levels of exosomal miR-214-3p are lower than those of patients without endometriosis (Zhang et al., 2021b). Injecting exosomes containing miR-214 mimics into a mouse model of experimental endometriosis decreased the expression of fibrosis-associated proteins (Wu et al., 2018). Intrauterine adhesion (IUA) caused by endometrial injury is one of the major causes of infertility in women. A promising treatment option for patients with severe intrauterine adhesions and infertility is topical administration of exosomes derived from adipose-derived mesenchymal stem cells (ADSC-exos) (Zhao et al., 2020). These manifestations of PCOS are oligo-anovulation, clinical and/or biochemical hyperandrogenism, and polycystic ovaries. Patients with polycystic ovary syndrome may have follicle development impairment due to elevated levels of estrogen and pregnenolone in follicular fluid; the mechanism is in part mediated by changes in the expression of HSD17B1, CYP19A1, and CYP11A1 in FF exosomes (Yu et al., 2021).

### Limitation

We only focused on exosomes in this study, as there have been published articles on microvesicles and their role in reproductive processes. The majority of the initial searches were conducted in the WOS database using the WOS classification and reproduction-related keywords; this may have led to the exclusion of papers pertinent to reproductive research that were not published in the classification. In addition, because this is a young and expanding field of research, we may have underestimated the impact of newly published studies on various analyses due to their low citation frequency, although some of the studies were published in high-quality journals. However, we believe that this study can still be utilized to convey the overall situation and general trends in the field.

## Conclusion

In reproduction science, exosomes have essential research value and application potential. Using CiteSpace and VOSviewer for visual analysis, the study of exosomes in reproduction research is developing significantly over the past years. The increasing number of reports published in international core journals is indicative of the importance of this topic. The United States, China, and Australia are the leading nations in such research; however, there is a need for increased cooperation and exchange between nations and institutions. All scholars should increase the greater collaborative effort and intersectionality of the research. In addition to focusing on fundamental research, we should concentrate on the application of the results and the investigation of exosomes in infertile patients. Current studies on exosomes in reproduction science focus on gamete development and fertilization, pregnancy, and reproductive cancer; these topics will be the focus of future studies.

## Data Availability

The original contributions presented in the study are included in the article/Supplementary Material, further inquiries can be directed to the corresponding author.
